# Biosynthesis of Benzohydroxamic Acid in *Streptomyces angustmyceticus*

**DOI:** 10.1021/acs.jnatprod.6c00526

**Published:** 2026-06-23

**Authors:** Jiangpeng Yu, Peibo Liang, Jie Wang, Qing Yang, Wei Li

**Affiliations:** † State Key Laboratory for Biology of Plant Diseases and Insect Pests, Institute of Plant Protection, 12661 Chinese Academy of Agricultural Sciences, Beijing 100193, China; ‡ Shenzhen Branch, Guangdong Laboratory for Lingnan Modern Agriculture, Shenzhen Key Laboratory of Agricultural Synthetic Biology, Genome Analysis Laboratory of the Ministry of Agriculture and Rural Affairs, Agricultural Genomics Institute at Shenzhen, Chinese Academy of Agricultural Sciences, Shenzhen 518124, China; § College of Life Sciences, South China Agricultural University, Guangzhou 510642, China; ∥ College of Life Sciences, Northwest A&F University, Yangling 712100, China

## Abstract

Benzohydroxamic acid (BHA) is an important hydroxamate scaffold with potential applications in medicinal chemistry and crop protection, but it has not previously been established as a natural product. Here, we report the discovery and biosynthetic characterization of BHA from *Streptomyces angustmyceticus*. A conserved TsnB-like gene cassette comprising *SaAmOx-AS*, *SaAmOx*, and *SaHAT* was identified by genome mining, indicating a potential capacity for hydroxamate formation and transfer. BHA was detected as a fermentation product of *S. angustmyceticus* and structurally confirmed by comparison with an authentic standard, compound purification, and NMR spectroscopic analysis. Benzoic acid was established as the aromatic precursor of BHA through deuterium-labeling experiments with benzaldehyde and cinnamic acid. SaHAT was identified as the key hydroxamate-transfer enzyme that directly converts benzoic acid, rather than benzoyl-CoA, to BHA using glutamyl hydroxamate as the hydroxamate donor in an ATP- and Mg^2+^-dependent reaction. BHA production was reconstituted in *Escherichia coli* by heterologous coexpression of SaAmOx-AS, SaAmOx, and SaHAT, confirming that these three enzymes form a minimal biosynthetic module. This work establishes BHA as a microbial natural product and provides a foundation for engineering hydroxamate-containing compounds.

## Graphical abstract

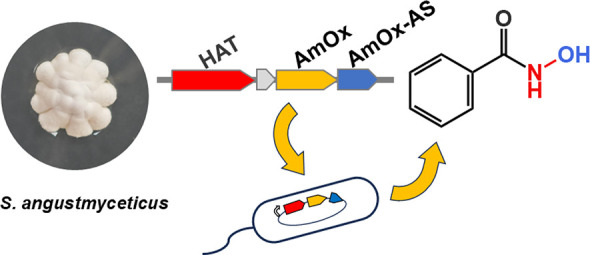

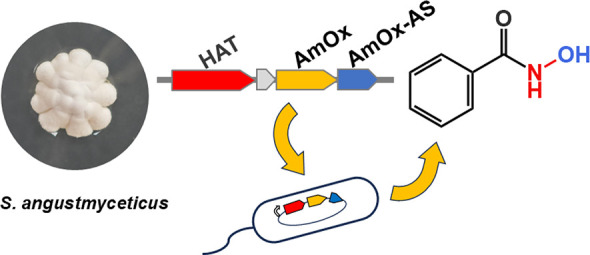

Benzohydroxamic acid (BHA), characterized by a terminal –CONHOH moiety, has been investigated in medicinal chemistry for its anticancer potential, frequently in conjunction with complementary structural elements.^1,2^ Notably, BHA has recently been identified as a specific inhibitor of chitin deacetylases (CDAs) in phytopathogenic fungi such as *Verticillium dahliae* and *Puccinia striiformis* f. sp. *tritici*, positioning it as a promising molecular pesticide for protecting against severe fungal diseases, including wheat stripe rust and soybean Verticillium wilt.^3^ The capacity of BHA is attributed to the chelation of Zn^2+^ ions within the active site of fungal CDAs by its hydroxamic acid group, leading to inactivation of CDA, and prevention of chitin deacetylation triggers a more effective plant immune response.^3,4^

Although the core structural elements, benzoic acid and hydroxamic acid groups, are prevalent in diverse microbes, no BHA has been reported as a natural product, and the commonly used BHA is obtained through chemical synthesis. The hydroxamic acid functionality is a significant and recurrent motif in microbial secondary metabolites, notably in siderophores^5,6^ and various antibiotics such as actinomycin^7^ and trichostatin A (TSA)^8^ from actinomycetes. The *TsnB* gene cluster in *Streptomyces* sp. *RM72* was deduced to govern TSA biosynthesis, critically involving *TsnB6&7* oxidation of l-glutamine to glutamyl hydroxamate and the subsequent *TsnB9* transfer of this hydroxamic acid moiety to trichostatic acid.^8^ Benzoic acid, featuring a benzene ring motif, serves as a crucial intermediate in pathways like enterocin biosynthesis, extensively characterized in *Streptomyces maritimus*, where it acts as a precursor to benzoyl-coenzyme A.^9^ Compounds such as salicylic acid and flavonoids activate immunity in plants.^10^ Aromatic compounds are biosynthesized predominantly via the isochorismate pathway,^11^ partly via the polyketide and terpenoid pathways.^12,13^

Nevertheless, despite the possibilities of biosynthesis of a hydroxamic acid group and a benzene ring, BHA has not yet been reported to be produced in nature. In this study, we identified BHA in *Streptomyces angustmyceticus* and proposed its biosynthetic pathway. *S. angustmyceticus*, a Gram-positive bacterium isolated from soil (phylum *Actinobacteria*, genus *Streptomyces*), is primarily recognized for producing angustmycin, a potent GMP synthesis inhibitor and plant cytokinin.^14,15^ *S. angustmyceticus* also effectively inhibits plant pathogens such as *Colletotrichum* sp. and *Curvularia lunata*, though its mechanism remains unknown.^16^ The findings provide a foundation for sustainable biosynthesis of BHA as a medicine and pesticide and may also give a clue to elucidate the mechanism of its biocontrol role.

## Results and Discussion

### Genome Mining of Hydroxamate Biosynthetic Genes in *S. angustmyceticus*

To identify potential hydroxamic acid-producing strains, we used the *TsnB* gene from *Streptomyces* sp. RM72 as a query in a tblastn search on NCBI. This search yielded 12 homologous proteins for *TsnB6*, while both *TsnB7* and *TsnB9* returned over 100 homologous proteins. The vast majority of these proteins originated from the phylum *Actinobacteria*, predominantly from the genus *Streptomyces*, with fewer hits from *Saccharothrix*, *Kibdelosporangium*, and *Amycolatopsis*.

For subsequent phylogenetic tree construction, we selected the top 21 (with >50% similarity) for TsnB9, 15 (with >40% similarity) for TsnB7, and all 12 homologous protein sequences (with >34%) for TsnB6. The analysis revealed that WP313772113 (SaHAT), WP313772112 (SaAmOx), and WP260640087 (SaAmOx-AS) from *S. angustmyceticus* exhibited high sequence homology to the RM72 *TsnB* genes, with similarities of 95.58% for *TsnB9*, 97.84% for *TsnB7*, and 86.98% for *TsnB6* (Figure 1A). The annotations and predicted functions of these core *TsnB* genes are summarized in Table 1.

**Figure 1:**
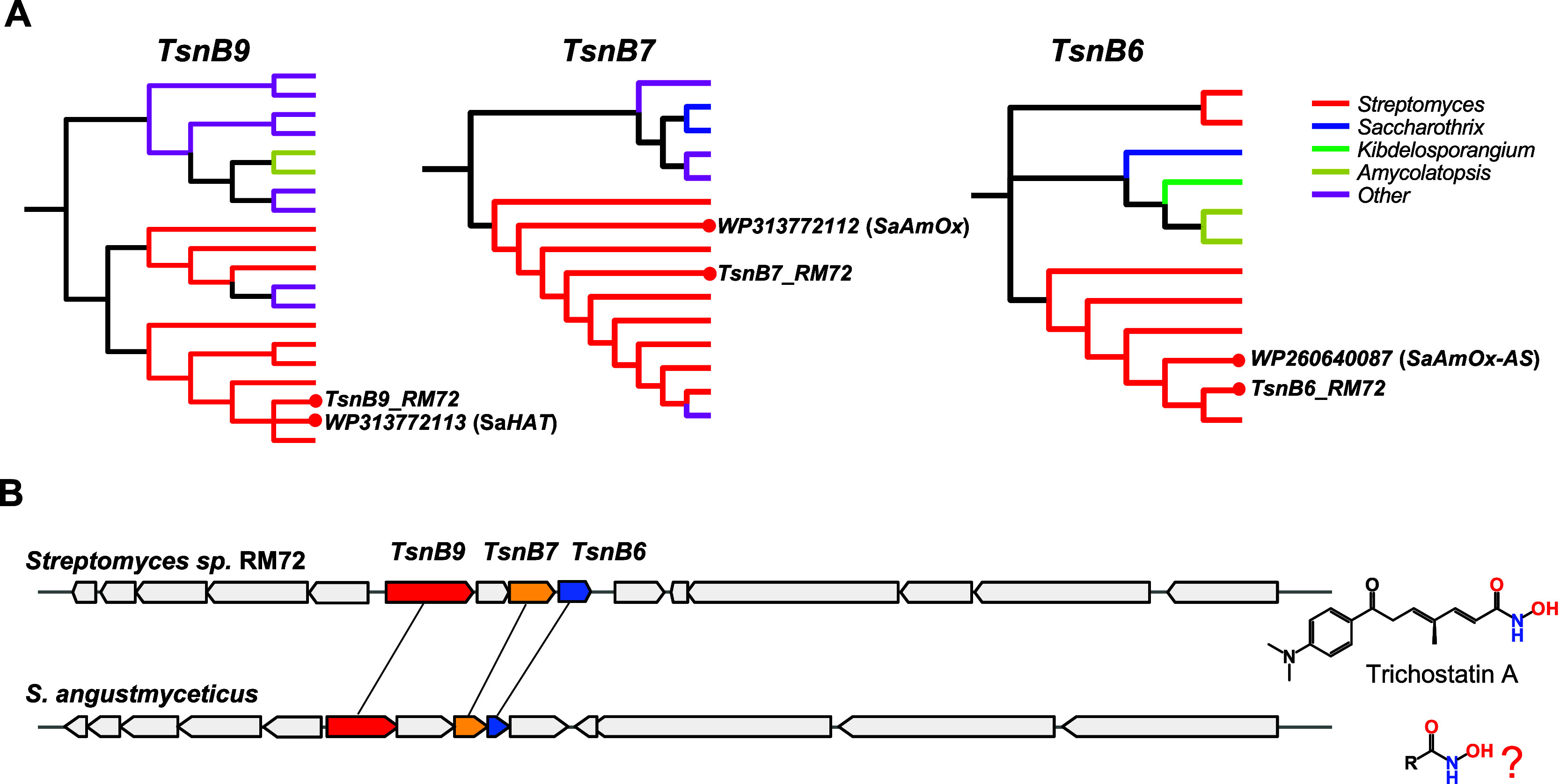
Identification of *TsnB* genes in *S. angustmyceticus*. (A) Phylogenetic analysis of *TsnB* genes from diverse microorganisms. Different colors represent strains from distinct genera. (B) Distribution of homologous TSA gene clusters in *S. angustmyceticus* and *S. RM72*.

**Table 1:** Annotation of the Core *TsnB* Genes within the Putative BHA BGC *S. angustmyceticus*

gene	protein	name	predicted function
*K7396_RS34045*	WP313772113	SaHAT (TsnB9_SA)	hydroxyamidotransferase
*K7396_RS34055*	WP313772112	SaAmOx (TsnB7_SA)	amidooxygenase
*K7396_RS34060*	WP260640087	SaAmOx-AS (TsnB6_SA)	amidooxygenase accessory subunit

Leveraging this phylogenetic insight, we analyzed the genomic region flanking these *TsnB* homologues in *S. angustmyceticus* to identify a putative hydroxamate biosynthetic gene cluster (BGC). As predicted by antiSMASH 8.0, this putative BHA BGC spans 54.5 kb and comprises 36 genes. Although it is notably larger than the canonical trichostatin A (TSA) BGC from *Streptomyces* sp. RM72, comparative genomic analysis demonstrated that all 14 core biosynthetic genes are highly conserved between these two clusters, maintaining high sequence similarity and synteny (Figures 1B, S1 and Table S1).

Within this conserved region, key biosynthetic genes include three *TsnB* homologues for hydroxamate moiety formation and type I polyketide synthase (PKS) genes for carbon scaffold assembly. Additional annotations reveal five ligases, three oxidoreductases, and one methyltransferase. Notably, conserved isochorismatase and isochorismate synthase genes suggest a potential role in supplying aromatic precursors. Furthermore, the presence of regulatory and transporter genes implies tightly controlled biosynthesis and efflux. This pronounced genomic conservation strongly suggests the potential for the presence of hydroxamate-containing compounds in *S. angustmyceticus*.

Comparative analysis of *TsnB*-containing gene clusters revealed that the *TsnB6/7/9* cassette is highly conserved across multiple actinomycete species, despite variations in overall cluster organization. This conservation suggests that these three enzymes constitute a minimal functional module for hydroxamate formation and transfer. The presence of additional tailoring enzymes and regulatory elements in different clusters may contribute to metabolic diversification but is not essential for the core hydroxamate-transfer steps in BHA assembly, provided the aromatic precursor is available.

Furthermore, genomic analysis using antiSMASH identified similar *TsnB* gene clusters in other actinomycetes, specifically *Streptomyces lydicus*, *Streptomyces* sp. NBC_00400, and *Saccharothrix syringae*. Although these clusters vary in size (containing 40, 42, and 32 genes, respectively), they all contain the three core *TsnB* genes. Specifically, *SaHAT* from *S. angustmyceticus* shares 100% and 97.45% amino acid identity with the *TsnB9* proteins in *Streptomyces* sp. RM72 and *S. lydicus* respectively. Similarly, SaAmOx shows 99.04% and 93.29% identity, and SaAmOx-AS shows 98.29% and 79.52% identity to their counterparts in these two strains. Notably, the methyltransferase gene *TsnB8*, usually located between *TsnB7* and *TsnB9* in *Streptomyces* species, is missing in *S. syringae*, resulting in a more compact *TsnB6/7/9* gene arrangement.

### Identification of Benzohydroxamic Acid (BHA) Produced in *S. angustmyceticus*

To experimentally validate the potential presence of hydroxamate suggested by genomic insights, *S. angustmyceticus* was collected from CGMCC (accession no. JCM4053) and cultured under standard laboratory conditions. On ISP2 agar, the strain formed colonies approximately 3 mm in diameter with irregular margins, displaying a white aerial mycelium over an off-white substrate mycelium, with no diffusible pigments observed (Figure S2A).

Metabolites were subsequently extracted from the fermentation cultures and analyzed via gas chromatography–mass spectrometry (GC–MS). Initial profiling of the *S. angustmyceticus* culture extract provided by a total ion chromatogram (TIC) demonstrated a rich array of metabolites (Figure S2B). Database matching mass spectral information against the NIST library led to the identification of 78 putative natural products. These compounds were categorized into diverse classes: alkaloids (36), aromatic compounds (10), amino acids (6), lactones (5), terpenes (3), polyketides (1), and others (17) (Figure S2C). This comprehensive metabolite screening particularly underscored the significant diversity of alkaloids, suggesting a robust capacity of *S. angustmyceticus* for producing nitrogen-containing secondary metabolites.

Subsequently, efforts were specifically directed toward the detection of BHA (Figure 2A). An extracted ion chromatogram (EIC) at *m*/*z* = 105.03345, representing a characteristic BHA fragment ion, was analyzed. To confirm the identity of this putative compound, its retention time and mass spectrum were subjected to comparison against an authentic BHA standard and a control strain, *Streptomyces* sp. S444 (lacking the homologous gene cluster). A prominent peak with a retention time of 20.23 min, identical to that of the BHA standard, was consistently observed in the *S. angustmyceticus* extract (Figure 2B). Crucially, a detailed comparison of the mass spectrometry fragmentation patterns revealed a high degree of congruence between the suspect peak from *S. angustmyceticus* and the authentic BHA standard (Figure 2C). The yield of BHA in *S. angustmyceticus* was quantified as 1.47 mg/L by comparison with an authentic standard. Collectively, the concordant chromatographic and mass spectral evidence unequivocally validates the production and accumulation of BHA by *S. angustmyceticus*. Furthermore, HPLC-HRMS analysis also detected trichostatin A (TSA) at 1.48 mg/L in the extracts (Figure S3). While these results suggest that the BGC produces polyketide-derived hydroxamic acid compounds, the comparable accumulation levels of both BHA and TSA suggest that the *TsnB*-like hydroxamate-transfer machinery in this strain may efficiently transfer the hydroxamic acid moiety to multiple acceptors.

**Figure 2:**
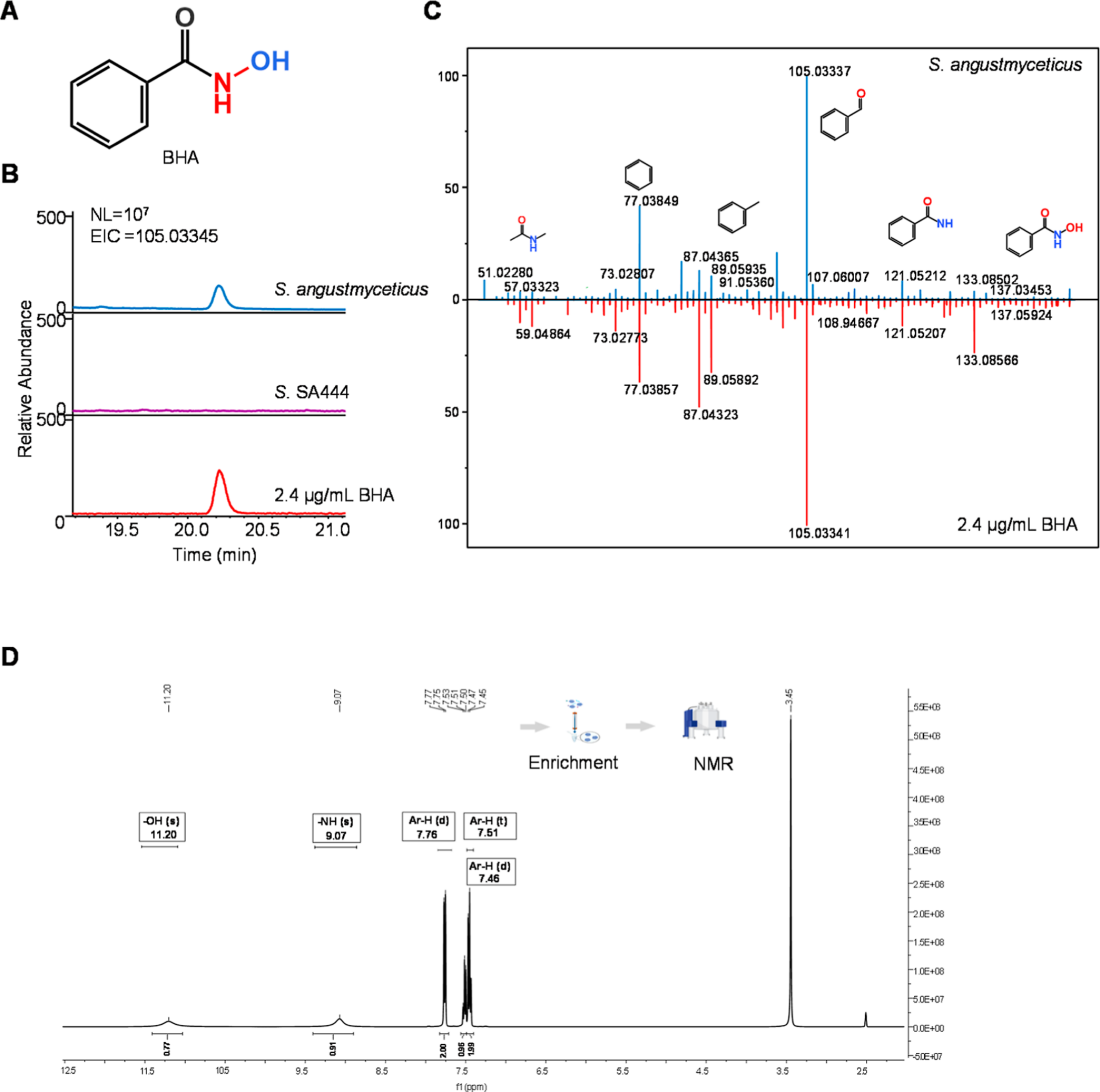
Identification of natural BHA from *S. angustmyceticus*. (A) Chemical structure of BHA. (B) GC–MS detection of BHA in *S. angustmyceticus* extract with S. S444 as negative control (EIC, *m*/*z* = 105.03345). (C) Comparison of mass spectra between natural BHA from *S. angustmyceticus* and BHA standard. (D) Purification and the ^1^H nuclear magnetic resonance (NMR) spectra analysis of BHA from *S. angustmyceticus*.

To further confirm the presence of BHA in *S. angustmyceticus*, a dedicated purification method was established. This method integrated standard *Streptomyces* compound isolation techniques with affinity chromatography (SO_3_–Fe^3+^). In total, 31.42 mg BHA was obtained, and analytical HPLC confirmed a purity of 98.70% (Figure S4). The complete material balance for this isolation process is summarized in Table S2. The purified compound was then subjected to nuclear magnetic resonance (NMR) spectroscopic analysis for definitive structural elucidation. The NMR spectroscopic data of the purified BHA exhibited excellent agreement with those reported for authentic BHA, unequivocally confirming its identity. The ^1^H NMR (400 MHz, DMSO-*d*_6_) spectrum showed resonances at δ 11.20 (s, 1H, OH), 9.07 (s, 1H, NH), 7.76 (d, *J* = 7.3 Hz, 2H, Ar-H), and 7.58–7.40 (m, 3H, Ar-H). The ^13^C NMR (101 MHz, DMSO-*d*_6_) spectrum displayed signals at δ 164.76 (C=O), 133.23, 131.62, 128.85, and 127.33 (aromatic C) (Figure S5). These data are consistent with literature values for BHA, confirming its structure. The raw NMR data files for BHA are provided in the Supporting Information.

### Isotope Feeding Confirms Benzoic Acid as a BHA Precursor

BHA, a conjugate of benzoic acid and a hydroxamic group, is proposed to be synthesized via a two-step pathway within *S. angustmyceticus*. The initial step is hypothesized to involve the oxidation of l-glutamine to glutamyl hydroxamate, potentially catalyzed by SaAmOx-AS and SaAmOx. Subsequently, SaHAT is proposed to mediate the transfer of the hydroxylamino group from glutamyl hydroxamate to benzoic acid, thereby generating BHA (Figure 3A).

**Figure 3:**
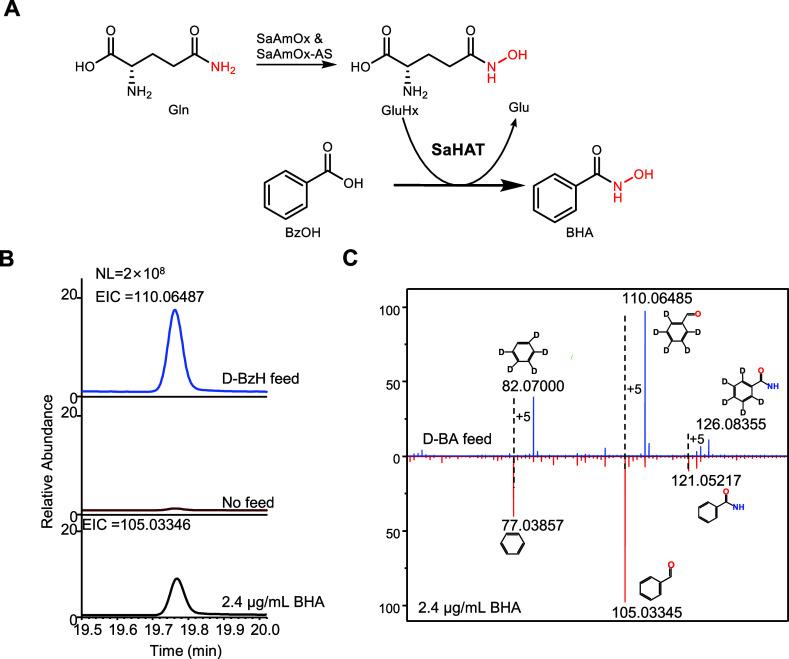
BHA biosynthetic pathway in *S. angustmyceticus*. (A) Proposed BHA biosynthetic pathway. BHA is generated by transferring the hydroxamic acid group from the donor glutamyl hydroxamic acid to the acceptor benzoic acid, catalyzed by SaHAT; glutamyl hydroxamic acid is produced from glutamate, catalyzed by SaAmOx and SaAmOx-AS. (B) Extracted ion chromatograms (EIC, *m*/*z* = 110.06487) of *S. angustmyceticus* culture fed with deuterated benzaldehyde. (C) High-resolution mass spectrometry comparison between deuterated BHA generated by feeding and a cold BHA standard.

Our hypothesis was further supported by feeding deuterated benzaldehyde at the benzenoid ring to *S. angustmyceticus*. Benzaldehyde was used instead of benzoic acid because it has more favorable water solubility and cell permeability and was readily oxidized to benzoic acid in vivo. High-resolution GC–MS analysis of the fed cultures unequivocally detected a characteristic peak at *m*/*z* 110.06487, corresponding to D5-BHA (Figure 3B). A comparative analysis with the unfed control revealed a consistent 5 Da increase in the mass of characteristic ions, precisely matching the expected shift for deuterium incorporation (Figure 3C).

As previously reported, benzoic acid is commonly derived from cinnamic acid in *Streptomyces*.^9^ Therefore, to investigate whether this route could be involved in BHA biosynthesis, we performed feeding experiments using deuterium-labeled cinnamic acid (d-CA) in *S. angustmyceticus*. GC–MS analysis revealed the formation of deuterium-labeled benzoic acid (73.9% incorporation) and BHA (42.9% incorporation), indicating that cinnamic acid can be converted into benzoic acid and subsequently incorporated into BHA (Figure 4). To further examine the upstream pathway, an expanded genomic search using multiple PAL sequences (including EncP from *Streptomyces* maritimus) only identified a histidine ammonia-lyase (HAL) with low sequence identity (37.76%). Additionally, feeding deuterated phenylalanine did not yield deuterated cinnamic acid, confirming the absence of a functional PAL in this strain (Figure S6). This suggests that a canonical PAL-dependent pathway may not be present in this strain, and the natural biosynthetic pathway of native benzoic acid remains unclear. Therefore, this evidence indicates that, at the feeding level, exogenously supplied cinnamic acid can be converted into benzoic acid and ultimately incorporated into BHA in *Streptomyces*.

**Figure 4:**
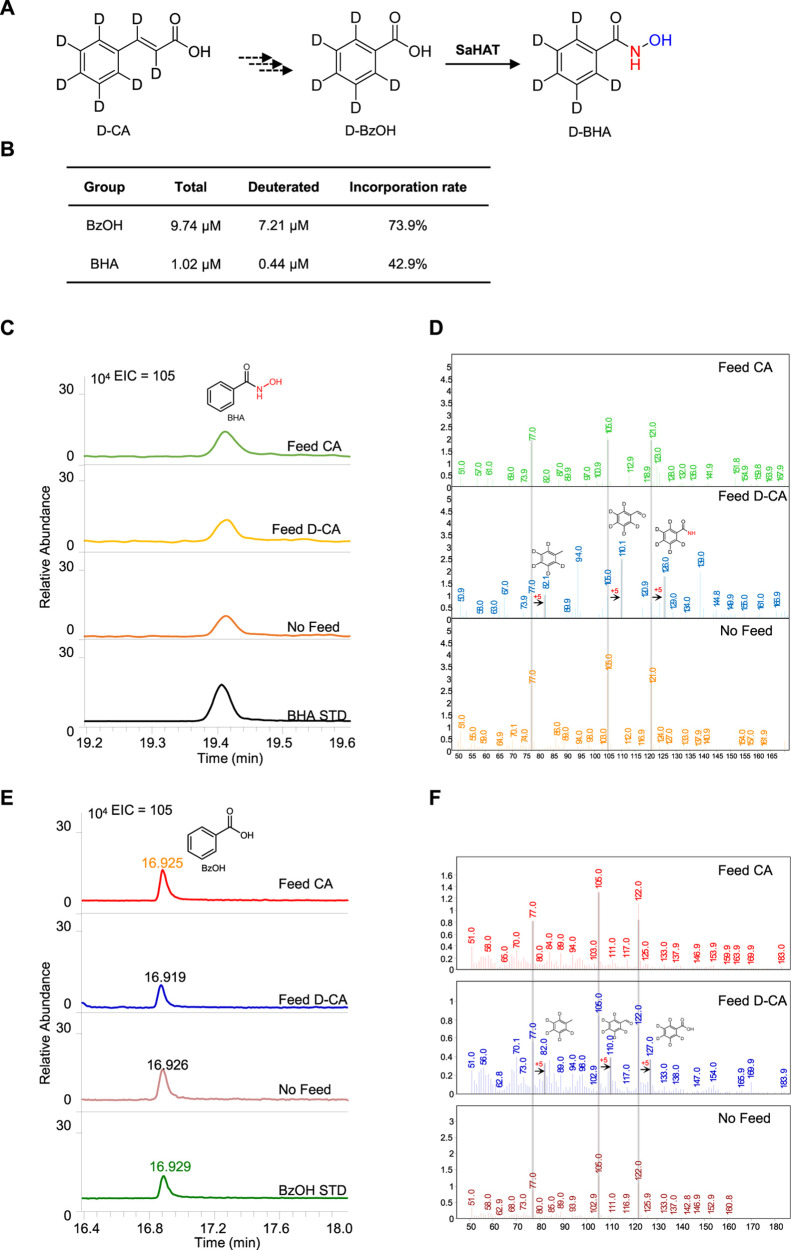
Deuterated cinnamic acid feeding of *Streptomyces angustmyceticus*. (A) The proposed conversion of deuterated cinnamic acid (d-cinnamic acid) to deuterated benzoic acid (d-BzOH) and deuterated benzohydroxamic acid (d-BHA). (B) Incorporation rate of deuterium in the feeding experiment. (C,D) Extracted ion chromatograms (EICs *m*/*z* = 105.0) (C) and MS spectra (D) showing BHA detection in different feeding groups. MS spectra highlight the characteristic mass shifts (*m*/*z* = 126.0, 110.0, 82.0) in the d-CA group, confirming efficient deuterated conversion. (E,F) EICs (*m*/*z* = 105.0) (E) and MS spectra (F) showing BzOH detection in different feeding groups. MS spectra highlight the characteristic mass shifts (*m*/*z* = 127.0, 110.0, 82.0) in the d-CA group, confirming efficient deuterated conversion.

### Biochemical Characterization of SaHAT

To identify the direct substrate for BHA biosynthesis, we performed in vitro biochemical assays with SaHAT. The enzyme was heterologously expressed in *Escherichia coli* and purified as an N-terminal MBP-tagged fusion protein (Figure 5A,B). The recombinant SaHAT converted benzoic acid to BHA, as confirmed by coelution with an authentic standard and matching MS spectra (Figures 5C,D, and S7). In contrast, no BHA was detected when benzoyl-CoA, a common *Streptomyces* metabolite,^9^ was tested as substrate (Figure 5C). Instead, SaHAT activates the free carboxyl group of benzoic acid in an ATP-dependent manner, a mechanism consistent with a recent report on a homologous hydroxyamidotransferase.^17^ These results indicate that benzoic acid is the direct substrate for SaHAT catalysis.

**Figure 5:**
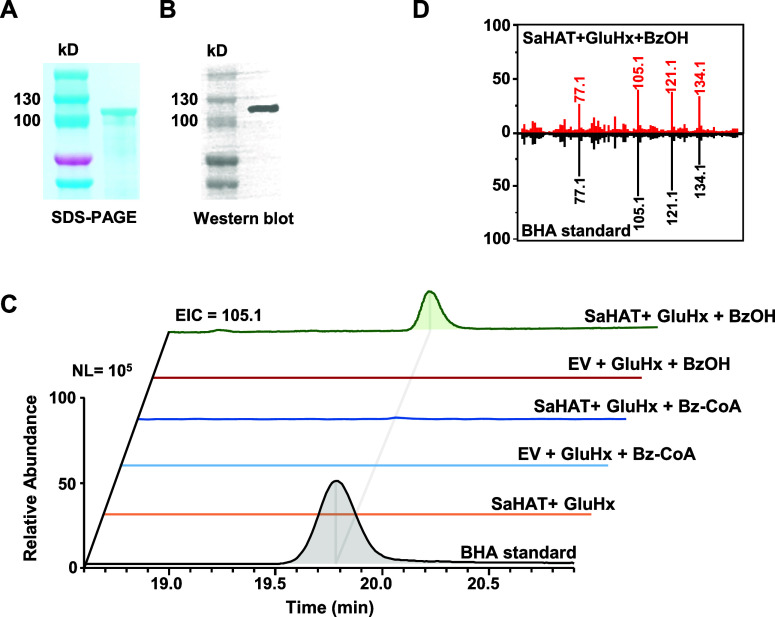
Biochemical characterization of SaHAT. (A) SDS-PAGE of the purified recombinant SaHAT. (B) Western blot of purified fusion protein. (C) GC–MS traces from in vitro enzymatic assays. BzOH: benzoic acid, Bz-CoA: benzoyl-CoA. (D) Mass spectra comparing the enzymatic product with the authentic BHA standard.

Notably, SaHAT utilizes benzoic acid rather than an activated benzoyl-CoA thioester, indicating a distinct biosynthetic strategy. The reaction was strictly dependent on Mg^2+^, and optimal activity was observed at pH 8.0 and 30 °C (Figure S8A–C). Steady-state kinetic analysis revealed Michaelis–Menten behavior for both substrates, with defined *K*_m_ and *k*_cat_ values, indicating efficient substrate recognition and turnover (Figure 6A–C). Together, these results suggest that SaHAT is the key enzyme mediating hydroxamate transfer in BHA biosynthesis.

**Figure 6:**
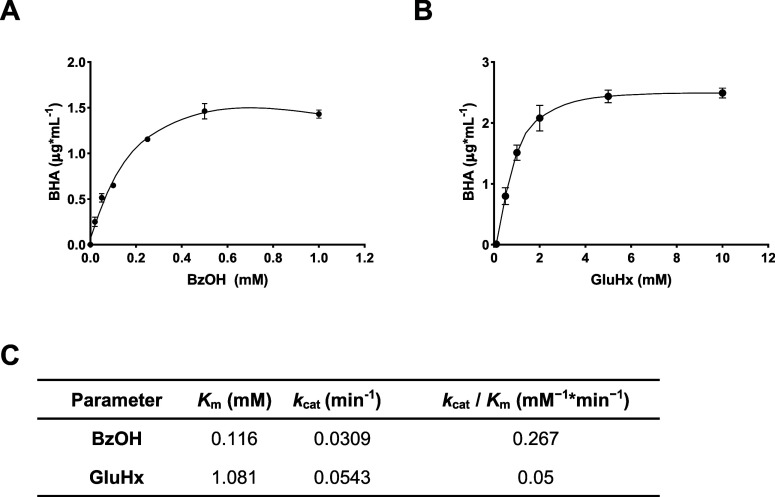
Kinetic analysis of recombinant SaHAT. (A) Measurement of BzOH *K*_m_ was achieved by varying the BzOH concentration (0–1.0 mM), SaHAT protein concentration 200 μg/mL, *N* = 3. (B) Measurement of GluHx *K*_m_ was achieved by varying the GluHx concentration (0–10 mM), SaHAT protein concentration 200 μg/mL, *N* = 3. (C) Kinetic parameters of SaHAT toward BzOH and GluHx.

### Validation of the BHA Biosynthetic Pathway by Heterologous Expression of *TsnB* Genes in *E. coli*

To further corroborate our hypothesis, the *SaAmOx-AS*, *SaAmOx*, and *SaHAT* gene sequences from *S. angustmyceticus* were codon-optimized (Table S4), and pRSF-SaHAT-SaAmOx–SaAmOx-AS was subsequently constructed for expression in *E. coli* BL21­(DE3) (Figure 7A). Induced *E. coli* cultures were then supplied with either benzaldehyde or a combination of benzaldehyde and glutamine. High-resolution GC–MS analysis showed that BHA was detected (EIC = 105.03345) in both benzaldehyde-fed samples and cofed samples (Figure 7B). To directly confirm the acid substrate in vivo as identified in our biochemical assays, parallel feeding experiments using benzoic acid were also conducted. GC–MS analysis showed that BHA was detected (EIC, *m*/*z* = 105.03345) in benzoic acid-fed samples (Figure S9). Multiple negative controls, including heat-inactivated cultures (boiled) and medium blanks (LB), showed no BHA production. Benzaldehyde feeding was also performed in *S. angustmyceticus* with rigorous controls. BHA levels increased markedly after feeding compared with nonfed cultures, whereas the boiled culture control showed no change (Figure S10). Given that *E. coli* endogenously synthesizes glutamine as a vital metabolite, BHA formation was observed even when only benzaldehyde was provided. Mass spectral comparison of these samples with a BHA standard confirmed congruent ion features (Figure 7C). Concurrently, the formation of glutamyl hydroxamate, catalyzed by SaAmOx-AS and SaAmOx, was detected by comparing both retention times and mass spectral data with those of an authentic standard (Figure S11). These results strongly support that SaAmOx-AS, SaAmOx, and SaHAT facilitate BHA biosynthesis, providing in vivo evidence for the proposed pathway.

**Figure 7:**
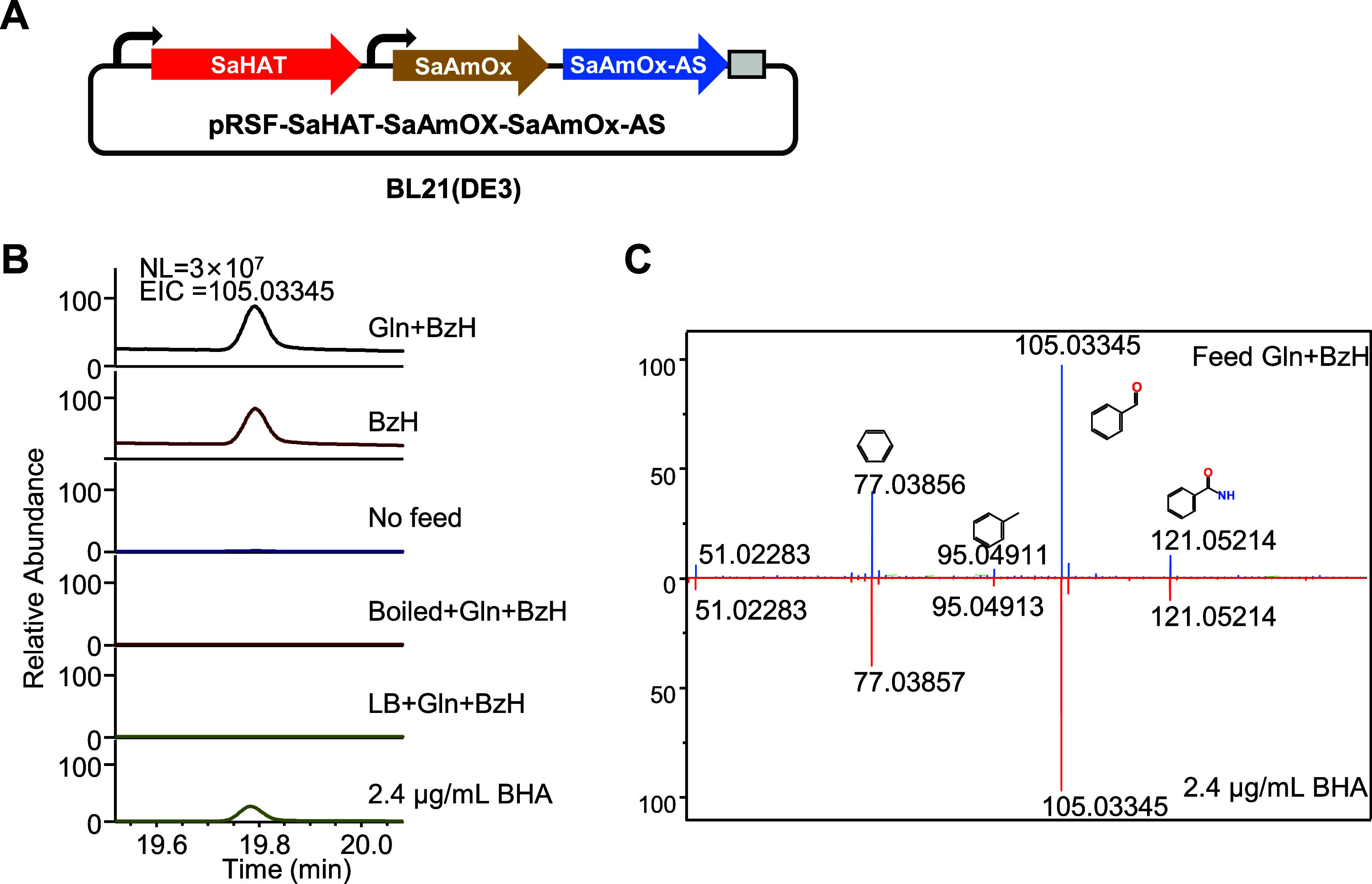
Validation of BHA biosynthesis through heterologous coexpression of SaHAT, SaAmOx, SaAmOx-AS genes in *E. coli*. (A) Schematic diagram of pRSF-SaHAT-SaAmOx–SaAmOx-AS expression plasmid. (B) EIC (*m*/*z* = 105.03345) from *E. coli* cultures heterologously expressing SaAmOx-AS, SaAmOx, and SaHAT after precursor feeding. Gln: glutamine; BzH: benzaldehyde. (C) High-resolution mass spectra of BHA in the fed sample, compared to the standard.

## Conclusions

In this study, we established benzohydroxamic acid (BHA) as a natural product produced by *S. angustmyceticus*. Genome mining, metabolite identification, isotope-feeding experiments, in vitro enzymatic assays, and heterologous reconstitution collectively support a biosynthetic pathway in which benzoic acid serves as the aromatic precursor and glutamyl hydroxamate provides the hydroxamate donor. The three TsnB homologues, SaAmOx-AS, SaAmOx, and SaHAT, constitute a minimal enzymatic module for BHA biosynthesis, with SaHAT directly transferring the hydroxylamino group to benzoic acid. These findings reveal a conserved hydroxamate-forming and -transferring gene cassette and suggest that BHA-like biosynthetic capacity may be more widespread in *Actinomycetes* than previously recognized. This work provides a basis for future studies on the distribution, evolution, and synthetic biology-based engineering of hydroxamate-containing natural products.

## Experimental Section

### General Experimental Procedures

High-resolution gas chromatography–mass spectrometry conditions: Thermo Scientific Orbitrap Exploris GC mass spectrometer, equipped with Thermo Scientific triplus RSH autosampler and Thermo Scientifics TRACE 1610 gas chromatograph, chromatographic column: Thermo Scientific TG-WAXMS (30 m × 0.25 mm × 0.25 μm). GC oven program: 80 °C (2 min), 10 °C/min to 230 °C (18 min hold). Injection (20:1 split); MS (EI, 70 ev, source 230 °C, full scan *m*/*z* 50–500). Data were processed using Xcalibur software.

GC-MSD conditions: Agilent Technologies 8890–5977B with Thermo Scientific TG-WAXMS column (30 m × 0.25 mm × 0.25 μm) was employed. GC column oven program: 80 °C (2 min), ramped to 230 °C at a rate of 10 °C/min (hold for 18 min). The GluHx detection program set the hold time to 37 min. The injection method was 20:1 split; mass spectrometry mode: EI, 70 eV, ion source temperature 230 °C, full scan *m*/*z* 50–500. Data analysis was performed using MassHunter 8.0.

High-resolution liquid chromatography–mass spectrometry conditions: Thermo Scientific Orbitrap Exploris 120 mass spectrometer, equipped with a liquid chromatograph, chromatographic column: C18. LC elution program (mobile phase A: water containing 0.1% formic acid; mobile phase B: acetonitrile containing 0.1% formic acid): 10% B to 90% B (0–4 min), 90% B (4–5 min hold), and 10% B (5–10 min hold for column equilibration). MS (ESI+ mode). Data were processed using Xcalibur software.

Unless otherwise specified, the concentration of the BHA standard is generally 2.4 μg/mL.

### Strain and Culture Conditions

*S. angustmyceticus* JCM 4053 (CGMCC), *Streptomyces* sp. S444 (Dr. X. Cheng, AGIS, CAAS) was used. Solid media were PDA and Gauze’s No. 1; liquid medium was TSB. Cultures were incubated at 30 °C (liquid at 220 rpm, 100 mL in 250 mL flasks for 5 days; solid for 3 days). For isotope benzaldehyde and cinnamic acid feeding, 0.5 mM (sterile filtered) was added to liquid cultures after 96 h, followed by 16 h incubation. Untreated cultures served as controls.

### Protein Purification and In Vitro Enzymatic Assays

Heterologous expression and purification of SaHAT: the gene encoding SaHAT was cloned into the pMAL vector to construct an expression cassette fusing the Maltose-binding protein (MBP) tag to the N-terminus of the target protein. The theoretical molecular weight of the resulting fusion protein was approximately 112 kDa. The recombinant plasmid was transformed into *E. coli* BL21­(DE3) competent cells for expression. A single colony was inoculated into 200 mL of Luria–Bertani (LB) broth and cultured at 37 °C with shaking at 220 rpm. When the optical density at 600 nm reached approximately 0.6, protein expression was induced by the addition of isopropyl β-d-1-thiogalactopyranoside (IPTG) to a final concentration of 0.5 mM. The cultures were then incubated at 16 °C for an additional 16 h. Cells were harvested by centrifugation at 4000 rpm for 10 min. The cell pellets were resuspended in 50 mL of lysis buffer (Tris-HCl, NaCl) and disrupted using a high-pressure cell homogenizer. The lysate was clarified by centrifugation, and the supernatant was applied to an amylose resin affinity column for purification. The target protein was eluted using an elution buffer containing 20 mM maltose. Protein concentration was determined using a commercial BSA protein assay kit, and the purified protein was stored in aliquots at −80 °C until further use.

In vitro enzymatic assays: the in vitro enzymatic reaction system was established based on the TSA biosynthetic assay protocol, as detailed in Table S3. The reaction mixtures were incubated at 30 °C for 30 min. To terminate the reaction, an equal volume of methanol was added to the mixture. The samples were then completely lyophilized using a freeze-dryer, resuspended in 200 μL of methanol, and processed for analysis following the sample preparation method described above.

### Optimization of Reaction Conditions

To determine the optimal catalytic conditions for SaHAT, various physicochemical factors were screened. The temperature dependence was evaluated from 20 to 40 °C and the optimal pH was determined using a series of buffers ranging from pH 5.0 to 10.0. Metal ion requirements were assessed by adding different ion salts Mg^2+^, Zn^2+^, Cu^2+^, Fe^3+^, Fe^2+^, Ca^2+^, Mn^2+^, and Na^+^ at a final concentration of 1 mM. Time-course (10 min to 4 h) and protein concentration gradient (1 to 1000 μg/mL) experiments were conducted to ensure the reaction occurred within the linear range. Determination of kinetic parameters (*K*_m_ and *k*_cat_) steady-state kinetic parameters for BzOH and GluHx were determined by varying the concentration of one substrate while keeping the other at a saturating level. For BzOH kinetics, the concentration of BzOH varied from 0.02 to 1.0 mM while GluHx was fixed at 10 mM. For GluHx kinetics, the concentration of GluHx varied from 0.1 to 10 mM while BzOH was fixed at 1 mM. The experimental data were fitted to the Michaelis–Menten equation using nonlinear regression analysis. The turnover number *k*_cat_ was calculated based on the *V*_max_ and the molar concentration of the enzyme (calculated from the molecular weight of 112 kDa).

### Heterologous Expression

The codon-optimized *SaHAT*, *SaAmOx*, *SaAmOx-AS* genes were cloned into the first and second MCS of the pRSFDuet-1 expression vector, respectively, and transformed into *E. coli* BL21­(DE3). A 100 mL LB culture, supplemented with 50 μg/mL kanamycin, was grown at 37 °C to an OD600 of approximately 0.6. Expression was induced with 0.5 mM IPTG, followed by overnight incubation at 16 °C. Subsequently, 0.5 mM benzaldehyde and 0.5 mM glutamine were added, and the bioconversion was carried out at 30 °C for 18 h. To prepare the heat-inactivated controls, the cultures were incubated in a 100 °C water bath for 20 min prior to substrate supplementation to ensure complete enzymatic inactivation.

### Metabolite Extraction

Five mL culture aliquots were lyophilized, resuspended in 10 mL methanol, vortexed, and sonicated for 40 min. After centrifugation (4000 rpm, 10 min), supernatants were concentrated to 1 mL (vacuum concentrator) and filtered (0.22 μm nylon membrane) before GC–MS analysis.

### Data Analysis

Compound identification was by retention time and mass spectra comparison with a BHA standard (≥99%, Yuanye Biotechnology) and the NIST library. Quantification of BHA used an external standard method. Metabolomic profiling used GC-rich software.

### Product Isolation and Purification

A total of 80 L of *S. angustmyceticus* fermentation broth was lyophilized. The resulting dried biomass was then extracted three times with 1 L of methanol via sonication. Following each extraction, the mixture was centrifuged to obtain the supernatant. The combined supernatants were concentrated to approximately 200 mL using a rotary evaporator. For preliminary purification and removal of nontarget compounds, the concentrated extract was loaded onto an affinity chromatography column packed with a sulfonic acid-modified resin. This resin, bearing ferric ions, was employed to leverage the metal-chelating properties of hydroxamic acids. The ferric hydroxamate chelate was eluted from the gel using approximately 30 mL of 25% aqueous methanol saturated with NaCl. The resulting chelate complex was then decomposed by titrating with about 10 mL of a freshly prepared 10% aqueous sodium dithionite solution until the color dissipated. Finally, the solution was lyophilized and redissolved in 1 mL of methanol for subsequent use.^18^ The BHA-containing fractions from the affinity chromatography were further concentrated.

The crude concentrate was subjected to purification via automated flash chromatography on a SANTAI SepaBean machine U100, using a prepacked 40 g silica column (40–63 μm) and a gradient elution of petroleum ether/ethyl acetate (10:1 to 1:1, v/v) at a flow rate of 20 mL/min. Fractions were collected based on UV absorption at 254 nm, and purity was confirmed by TLC, GC–MS, and NMR. The identity of BHA was confirmed by NMR spectroscopy.

NMR: the NMR standard concentration used was 80 mg/mL. ^1^H NMR (400 MHz, DMSO-*d*_6_): δ 11.20 (s, 1H), 9.07 (s, 1H), 7.76 (d, *J* = 7.3 Hz, 2H), 7.58–7.40 (m, 3H). ^13^C NMR (101 MHz, DMSO-*d*_6_): δ 164.76, 133.23, 131.62, 128.85, 127.33.

### Statistical Analysis

GraphPad Prism 8.0 was used for statistical analysis. BHA production differences between control and treated groups were assessed by Student’s *t* tests (*P* < 0.05 significant, *P* < 0.01 highly significant). *n* values are in the figure legends. Data are presented as mean ± SD.

## Supplementary Material







## Notes

raw NMR data of BHA (ZIP)
GeneBank files (ZIP)
Annotation of the BHA biosynthetic gene cluster (Table S1, PDF); complete material balance for purification (Table S2, PDF); in vitro SaHAT reaction mixture (Table S3, PDF); codon-optimized sequences (Table S4, PDF); morphology, metabolite profiles, and HPLC/NMR spectra (Figures S1–S6, PDF); enzymatic assays and additional feeding data (Figures S7–S10, PDF) (PDF)
