# Supplementary figures and images for: Biosynthesis of Benzohydroxamic Acid in Streptomyces angustmyceticus
Source: J Nat Prod. 2026 Jun 23;89(7):2133–42. doi: 10.1021/acs.jnatprod.6c00526 (PMC13418202; doi:10.1021/acs.jnatprod.6c00526)

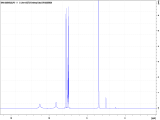

Supplement: Supplementary file 1 [file np6c00526_si_001.zip › BHA-NMR/H/pdata/1/thumb.png]
