# Supplementary material for: Biosynthesis of Benzohydroxamic Acid in Streptomyces angustmyceticus
Source: J Nat Prod. 2026 Jun 23;89(7):2133–42. doi: 10.1021/acs.jnatprod.6c00526 (PMC13418202; doi:10.1021/acs.jnatprod.6c00526)
Supplement: Supplementary file 3 [file np6c00526_si_003.pdf]

Supporting information for

Biosynthesis of benzohydroxamic acid in Streptomyces angustmyceticus

Jiangpeng Yu [a,b,c], Peibo Liang [a,b], Jie Wang [b,d], Qing Yang [a,b *], Wei Li [b *]

[a] State Key Laboratory for Biology of Plant Diseases and Insect Pests, Institute of Plant Protection, Chinese Academy of Agricultural Sciences, 100193, Beijing, China

[b] Shenzhen Branch, Guangdong Laboratory for Lingnan Modern Agriculture, Shenzhen Key Laboratory of Agricultural Synthetic Biology, Genome Analysis Laboratory of the Ministry of Agriculture and Rural Affairs, Agricultural Genomics Institute at Shenzhen, Chinese Academy of Agricultural Sciences, Shenzhen 518124, China

[c] College of Life Sciences, South China Agricultural University, Guangzhou 510642, China

[d] College of Life Sciences, Northwest A&F University, Yangling 712100, China

*** Corresponding author:**

Qing Yang - State Key Laboratory for Biology of Plant Diseases and Insect Pests, Institute of Plant Protection, Chinese Academy of Agricultural Sciences, 100193, Beijing, China; Email: qingyang@caas.cn

Wei Li - Shenzhen Branch, Guangdong Laboratory for Lingnan Modern Agriculture, Shenzhen Key Laboratory of Agricultural Synthetic Biology, Genome Analysis Laboratory of the Ministry of Agriculture and Rural Affairs, Agricultural Genomics Institute at Shenzhen, Chinese Academy of Agricultural Sciences, Shenzhen 518124, China; Email: liwei11@caas.cn

Table of Contents

Table S1. Annotation of the BHA biosynthetic gene cluster S1

Table S2. Summary of the material balance through the BHA purification steps......S2

Table S3. Composition of the *in vitro* SaHAT reaction mixture................................S2

Table S4. Optimization of TsnB gene sequences..S3 - S4

Figure S1. Organization of the predicted biosynthetic gene cluster located at 7,757,567 - 7,813,337 nt. on *Streptomyces angustmyceticus* strain JCM4053 genome S5

Figure S2. Metabolites in *S. angustmyceticus*..S5

Figure S3. Identification of natural TSA from *S. angustmyceticus*............................S6

Figure S4. HPLC analysis of purified BHA. S7

Figure S5. The ^{13}C Nuclear Magnetic Resonance (NMR) spectra of purified BHA...S8

Figure S6. GC-MS detection of cinnamic acid in *S. angustmyceticus* extract............S8

Figure S7. Gas chromatograms of *in vitro* enzymatic assays with SaHAT.................S9

Figure S8. Biochemical characterization of recombinant SaHAT...............................S9

Figure S9. Detection of BHA in *E. coli* cultures heterologously expressing SaAmOx-AS, SaAmOx, and SaHAT after precursor feeding..S9

Figure S10. Representative GC-MS analysis of BHA production in *S. angustmyceticus*...S10

Figure S11. Gas chromatography-mass spectrometry detection results of GluHx...S10

Table S1. Annotation of the BHA biosynthetic gene cluster in *Streptomyces angustmyceticus* and comparative analysis with *Streptomyces sp*. RM72.

Streptomyces angustmyceticus	location			identication	RM72	identity
K7396_RS33995	7758611	7759208	+	LysE family translocator		
K7396_RS34000	7759207	7759531	+	acyl carrier protein		
K7396_RS34005	7759550	7760942	+	TrpB-like pyridoxal phosphate-dependent enzyme		
K7396_RS34010	7760968	7762882	+	FAD/NAD(P)-binding protein		
K7396_RS34015	7762893	7764564	+	AMP-binding protein		
K7396_RS34020	7764649	7765198	-	cysteine hydrolase family protein	AYM48691.1	97
K7396_RS34025	7765194	7766010	-	aminotransferase class IV	AYM48692.1	94
K7396_RS34030	7766003	7767479	-	AMP-binding protein	AYM48693.1	97
K7396_RS34035	7767472	7769602	-	aminodeoxychorismate synthase component I	AYM48694.1	97
K7396_RS34040	7769641	7770883	-	3-deoxy-7-phosphoheptulonate synthase	AYM48695.1	96
K7396_RS34045	7771258	7773094	+	asparagine synthase (glutamine-hydrolyzing)	AYM48696.1	98
K7396_RS34050	7773109	7773811	+	methyltransferase	AYM48697.1	97
K7396_RS34055	7773838	7774780	+	diiron oxygenase	AYM48698.1	98
K7396_RS34060	7774871	7775399	+	hypothetical protein	AYM48699.1	93
K7396_RS34065	7776062	7777118	+	helix-turn-helix transcriptional regulator	AYM48700.1	96
K7396_RS34070	7777150	7777531	-	winged helix-turn-helix transcriptional regulator	AYM48701.1	98
K7396_RS34075	7777566	7783584	-	type I polyketide synthase	AYM48702.1	95
K7396_RS34080	7783588	7789033	-	type I polyketide synthase	AYM48704.1	91
K7396_RS34085	7789050	7793337	-	type I polyketide synthase	AYM48705.1	93
K7396_RS34095	7794109	7794736	-	SAM-dependent methyltransferase		
K7396_RS34100	7794823	7795450	+	helix-turn-helix domain-containing protein		
K7396_RS34105	7795499	7796477	-	helix-turn-helix transcriptional regulator		
K7396_RS34110	7796634	7797579	-	VOC family protein		
K7396_RS34115	7797620	7798748	-	FAD-dependent monooxygenase		
K7396_RS36050	7798919	7799435	-	flavin reductase family protein		
K7396_RS34120	7799429	7800644	-	styrene monooxygenase/indole monooxygenase family protein		
K7396_RS34125	7800718	7801939	-	styrene monooxygenase/indole monooxygenase family protein		
K7396_RS34130	7802456	7803437	+	AraC family transcriptional regulator		
K7396_RS34135	7803471	7804653	-	acyl-CoA dehydrogenase family protein		
K7396_RS34140	7804773	7805403	-	GNAT family N-acetyltransferase		
K7396_RS34145	7805617	7806646	+	S66 family peptidase		
K7396_RS34150	7806897	7807713	+	SDR family NAD(P)-dependent oxidoreductase		
K7396_RS34155	7808076	7809582	-	IPT/TIG domain-containing protein		
K7396_RS34160	7809578	7810451	-	YncE family protein		
K7396_RS34165	7810843	7811968	-	homogentisate 1,2-dioxygenase		
K7396_RS34170	7812176	7813133	+	LysR family transcriptional regulator		

Table S2. Summary of the material balance through the BHA purification steps.

Step	Total volume	Target content, mg	Yield %
Raw Culture	80 L	117.63	-
Crude Extract	0.2 L	78.31	66.57
Immobilized Iron Affinity	1 mL	35.68	45.56
RPLC	-	31.42	88.06

Table S3. Composition of the *in vitro* SaHAT reaction mixture.

Components	Concentration （100μl）
Tris-HCl (pH 8.0)	100 mM
BzOH, BzH ,Bz-CoA	0.1 mM
GluHx	1 mM
ATP	1 mM
MgCl2	5 mM
SaHAT	1 mg / ml

Table S4. Optimization of TsnB gene sequences.

Gene	Sequence
SaHAT	ATGTGCGGCGTTGCGGGCTGGGTTAGCTTTCGTCAGGAC<br>CTGAGCCACGAAGAAAACATCCTGGCGGGCATGACCAAC<br>AGCATGACCTGCCGCGGTCCGGATGCGAGCGGCCAGTG<br>GCTGAGCCGCCACGCGGCTCTGGGCCACCGTCGCCTGA<br>GCATCATTGACCTGCCGGGTGGCACTCAGCCGATGACCG<br>TTGATACCCCAGGCGGCCCGGTGACCATGAGCTACAGTG<br>GTGAAACCTACAACTTCGTGGAACTGCGTGATGAACTGC<br>GTAAACGCGGCCATACCTTTCGCACCCGCTCTGATACCG<br>AAGTGGTGCTGCGTGGTTACCTGGAATGGGGCGCGGCG<br>ATCGCGGAACGTATGGTTGGCATGTGCGCGATCGCGATC<br>TGGGACAGCCGCTATGAACGTCTGACCCTGATCCGTGAC<br>CGTATGGGCACCAAACCGATGCACTACAGCCGTACCAAA<br>GATGGCCTGCTGTTCGGCAGCGAACCTAAAGCTATCCTG<br>GCGCACCCGGATGCGAAACCGGTGGTTGATATGGAAGG<br>CATGCGTCAGCTGTTCAGCTTCTTCACCAGCTCTGAAAAC<br>GCAGTTTGGGCGGGCATGAAAGTTATGACTCCGGGCACC<br>GTTATTGAATTCGACCGTAACGGCCTGCGTGAACACACCT<br>ACTGGCAGCTGAGCGCCGAAGAACATACCGATGATCTGG<br>ATACCACCGTGGCGCGTGTGCGTCAGATGGTTGAAGATA<br>ACGTCCGCCACGAACTGGTGGCTGACGTGCCGCTGGGT<br>CTGCTGCTGTCCGGTGGTCTGGACTCTTCTGCGCTGGCT<br>GGTATTGCATCCCGTCACCTGACCGCGAAAGGTGATCGC<br>GCACGCACCTTCAGCGTGGACTTTCCAGGCCAGTCTGAA<br>AACTTCCAGCCGCACGAAATGGCTGATAGCGCTGATGCG<br>CCGTATGCAAAAGAAATGGCCGCGCACATTGGTTCTGAA<br>CATCACGATATTGTTCTGGATCATCGTCGTCTGAGCGACC<br>CGGACCTGCGCCGCTCTGTTGTTGCTGCGTGGGATCTGC<br>CGTGGGGCATGGGTGACATTAATGGTTCCATGTACCTGC<br>TGTTCAAAGCAGTTCGTGAACACGTTACCGTGGCACTGTC<br>CGGTGAAGCAGCGGACGAAATTTTCGCCGGCCACGTGTG<br>GCACCAGTCTAAAGCGGCTCGTTACGGTGGCACCTTCCC<br>GTGGCACACTACCTGGCTGAAACGTGTTGATTGCTCCGC<br>GTATCTGACCGGCGAGTTCAACGCGGCGCTGGATTCTGA<br>CGCGTACACCGCGGATCGTTTCCAGGAAGCTACCGCGC<br>GTGTGCCATACCTGGACGGCGAAGATGAAGAACAGCGTA<br>TGTACCGTCGCAGCCTGCACTTAGGCCTGAACCACTTTAT<br>GCGTGTACTGGAAGATCGCGTGGATCGTATGGCGATGGC<br>AGTTGGTCTGGAAACCCGTGTTCCGTTTTGCGATTACCGT<br>CTGGCGCAGTATCTGTACAACGTTCCGTGGACCATGCAG<br>ACTTTCGATGGCCGTGAAAAAAGCCTGCTGCGCGCTAGC<br>GTTAAAGATGTTGTGACTCCGAGCGTCGTGGAACGTCGT<br>AAAAGCCCGTACCCGAGCACCCAGGACACCCTGTACGTT<br>GGTGCGCTGCAGGAACAAGTGAAAATTCTGCTGAAAGAA<br>CCGAGCTCTCCGGTTTTCGATCTTTTTGACCGTAGCAAAC<br>TGGCAGAAGCGGCGGAACTGAGCCCGCAGCAGATCGCG<br>GGTGCGCCGCGTGCGGCGTTCGAAAAAGCGCTGGATCT<br>GGCTGTTTGGTTCGAAATCCGTAACCCGGAACTGCGTTAT<br>TAA

SaAmOx	ATGAACCGTACCGAAGAATCTGGTTATCGTAGCCTGTTCA<br>AACAGTGGGATAGCCGTTCTTGGGTTCGTAGCAAACCGC<br>GTCGCAACGGCGCATTCGCGAGCGGCCTGCACTACTTCA<br>GCCCGGATCTGTGCCCGCTGCTGGCGCACCCGGAAGTG<br>CGTGCGGCGCCGGCTCAGGTTCGTGAAGAAATCCTGGTT<br>CACTCTCTGTATGTTTACCTGGAATTCACCGTTCAGCTGG<br>AACTGGGTCCGGTTAACGAAACCTGCCTGCTGCTGCACA<br>GCCCGGATTTCTGCCCGTGGCTGCCGGCGGCAATGAAA<br>GAGGATCTGCTGCGTATCTACACCGATGAAGCTGCGCAC<br>GCGGAAATGAGCCACACCCTGCTGGCGACCGTTCGTGAC<br>CACACCGGCGTGGAACCGGTTCTGCACCGTCCGTACTTC<br>CTGCAGGAACTGTCCCGTCTGTACGCGGCGGAACTGCC<br>GGTTTACCGTCCGCTGGTTAAACTGTTCTTCAGCATCGTG<br>TCTGAAACCCTGATCACCGGCAGCCTGACCAAACTGCCG<br>AAAGATCCGTCTGTTCAGCAGGCTGTTCGTGAACTGGCG<br>GCTGATCACGCGACCGATGAAGGTCTGCACCACGCGTAT<br>TTCCGTCGTCTGTTTAAATCCCTGTGGCCGAAAATGCCGG<br>CGCCGCTGCAGACCAAAATCGGCGCTCTGCTGCCGGAAA<br>TCATCCTGGCGTTCCTGCGTCCGGATGAAGCGGCGATGA<br>CCCGTACCCTGGCGGGCTACCCGGAAATCTTCGAAGATC<br>CGGCGCGCGTTGTTGCGGAAACCGTTGAACTGCCGCGT<br>GTTCGTGGTAGCCTGGTTGATAACGCGGCGCCGACCCTG<br>CGTATGCTGGCGCAGGAAGGCGTTTTCTCTGATCCGGTT<br>ATTTCTGCAGCATTTGATAAACATGGTCTGCGTCAGCACG<br>CTTAA
SaAmOx-AS	ATGATCAACGATTACATCCTGGCGCTGCTGAGCGCGCCG<br>CCGGCGAACCCGATGGAATTCGCAGATGGCCAGCCGGC<br>GCTGTGGATGGATAACCTGCGTAAATGGCTGGATCGTGC<br>GGCGGAACTGGATCACTCCTTCCGTTTCGATGAACCGGT<br>TCCGGATCAGGATAACCGTCGTCTGCGTTTACGTACCCG<br>TCTGCCGCTGGCGAGCACCACCGATTTCGTTATCCACAC<br>CAGCGCTGGTTTCGATCGTCCGGGTCCGAGCTGGGCGG<br>GTCCGGCGGATTGCGATCTGGTTGTTCACTTCCTGTGCA<br>TGACCGATCGCCCGGCGGCGCCGCAGTCTCCGCCGCGT<br>GAAGATCCGTGCACCCCGTGGGCGGGTCGTGAAGGTCT<br>GCTGGTTGTTGAACCGCGTTGGGCGGCGGCGGTTCTGAT<br>GGCGCACGAAGCGCCGCTGTTCTGGGGTGCGAGCGCGC<br>GTGAAGAAAGCAAAAGCCTGCTGGATAGCCCGCGTAGCG<br>GTGCGGGTGGTGCTGGCGGTGCGTAA

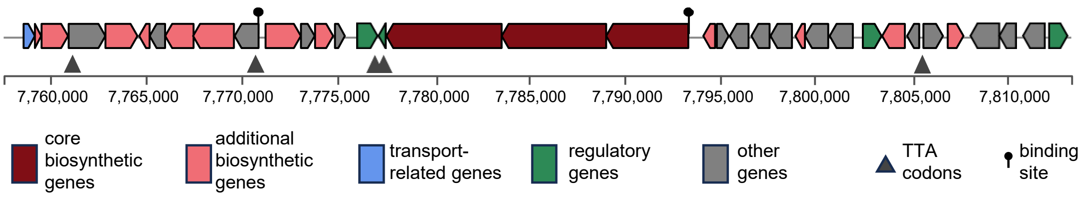

Figure S1. Organization of the predicted biosynthetic gene cluster located at 7,757,567 - 7,813,337 nt. on *Streptomyces angustmyceticus* strain JCM 4053 genome. Arrows indicate the direction of transcription, and genes are color-coded based on their predicted function: core biosynthetic enzymes (dark red), additional biosynthetic genes (red), transport-related genes (blue), regulatory genes (green), other genes (grey). The specific gene annotations within the cluster include: *K7396_RS34075, K7396_RS34080, K7396_RS34085* (type I polyketide synthase), *K7396_RS34045* (asparagine synthase (glutamine-hydrolyzing)), *K7396_RS34055* (diiron oxygenase).

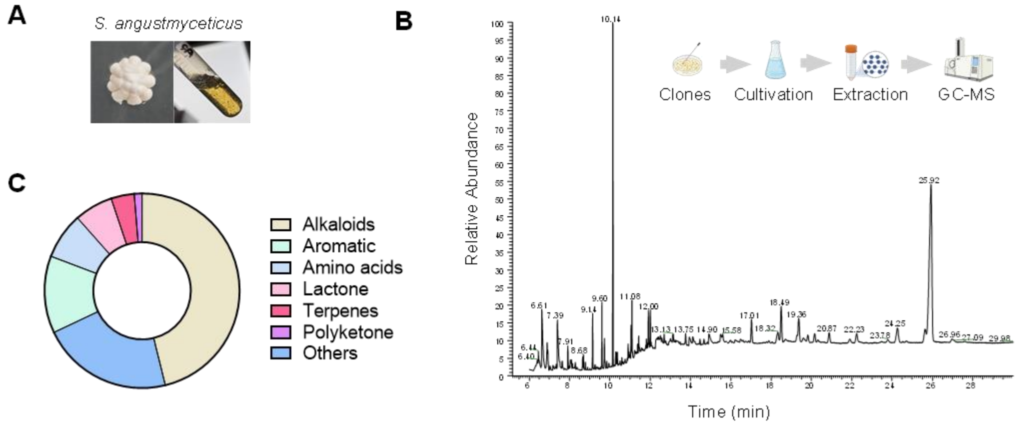

Figure S2. Metabolites in *S. angustmyceticus.*

A. Morphology of *S. angustmyceticus* on ISP2 agar (left) and in TSB broth (right). B. Total ion chromatogram (TIC) of *S. angustmyceticus* metabolites detected by GC-MS. C. Annotation and classification of natural products from *S. angustmyceticus*.

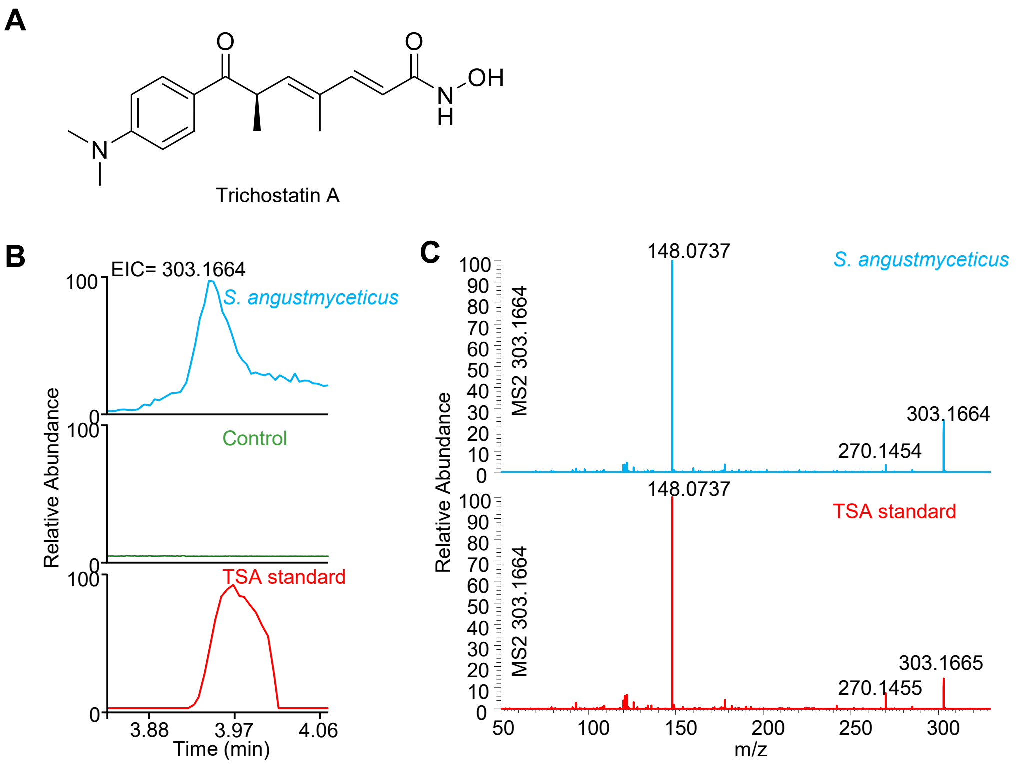

Figure S3. Identification of natural TSA from *S. angustmyceticus.*

A. Chemical structure of trichostatin A (TSA). B. HPLC-HRMS detection of TSA in *S. angustmyceticus* extract (EIC, m/z=303.1664). C. Comparison of mass spectra between natural TSA in *S. angustmyceticus* and TSA standard.

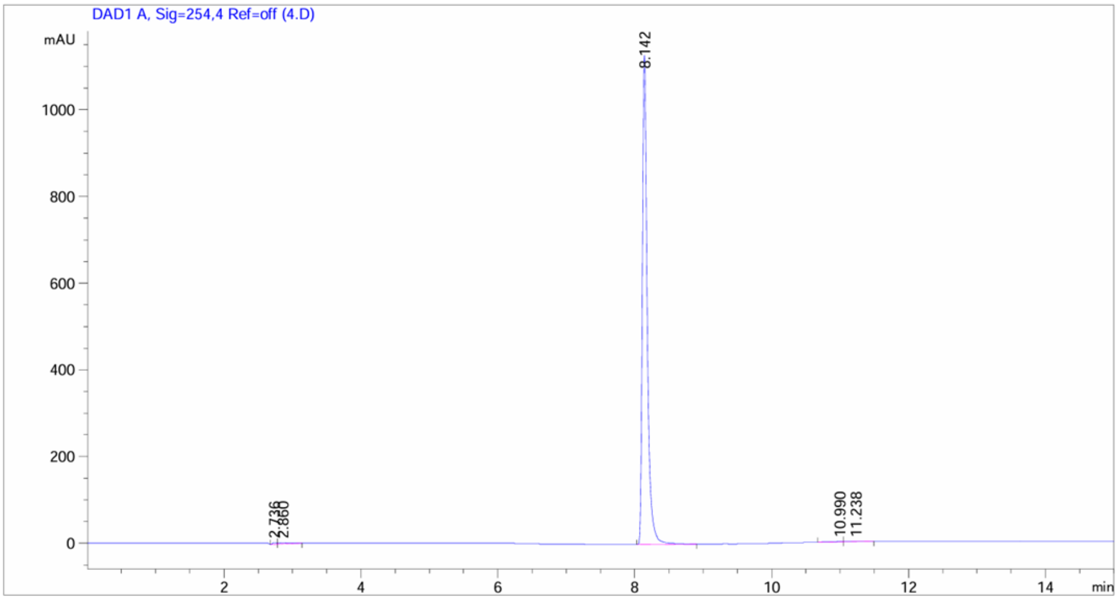

Peak	Retention time (min)	Peak width (min)	Peak area (mAU*s)	Peak height (mAU)	Peak area (%)
1	2.736	0.0626	14.42357	3.61192	0.2566
2	2.860	0.1561	29.10560	2.49537	0.5179
3	8.142	0.0732	5546.96338	1130.93164	98.6980
4	10.990	0.1167	8.97735	1.03971	0.1597
5	11.238	0.2434	20.66718	1.15159	0.3677

Figure S4. HPLC analysis of purified BHA. The peak area of BHA is approximately 98.7%.

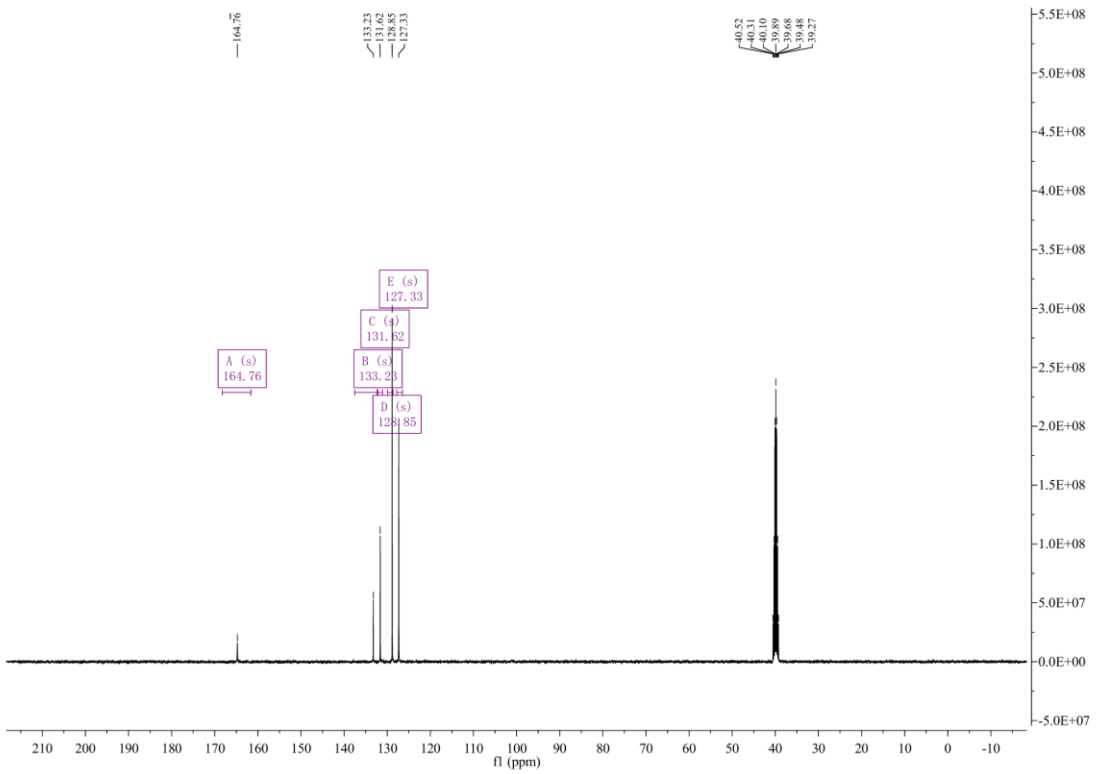

Figure S5. The ^{13}C Nuclear Magnetic Resonance (NMR) spectra of purified BHA.

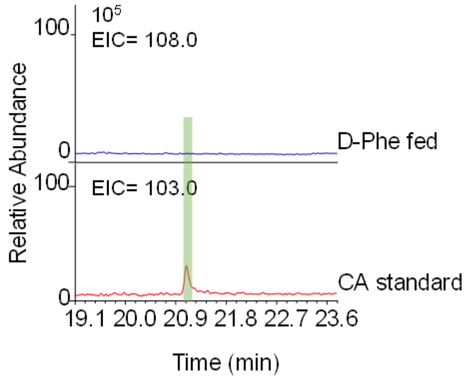

Figure S6. GC-MS detection of cinnamic acid in *S. angustmyceticus* extract (EIC, m/z=108.0; 103.0).

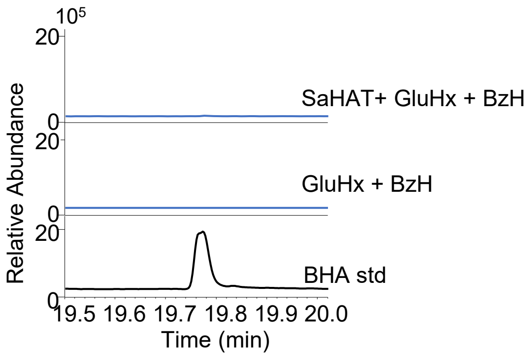

Figure S7. Gas chromatograms of *in vitro* enzymatic assays with SaHAT. BzH: Benzaldehyde.

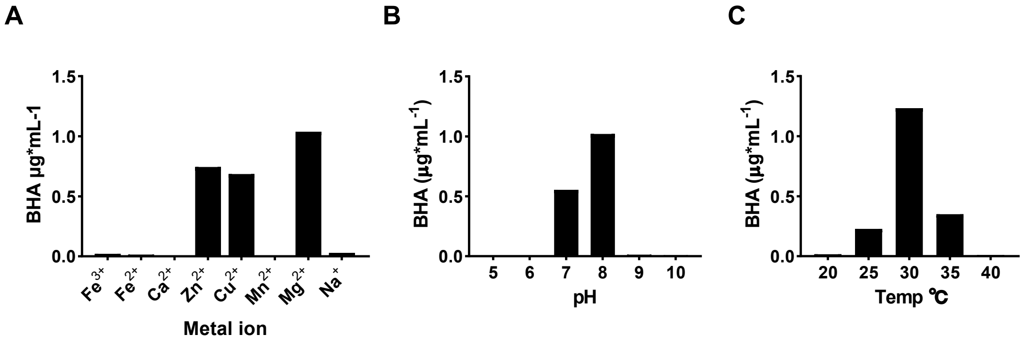

Figure S8. Biochemical characterization of recombinant SaHAT.
A. Effects of various metal ions (1 mM) on the enzymatic activity. B. Effect of pH on the biosynthesis of BHA (pH 5.0–10.0). (C) Effect of temperature on the enzymatic activity (20–40℃).

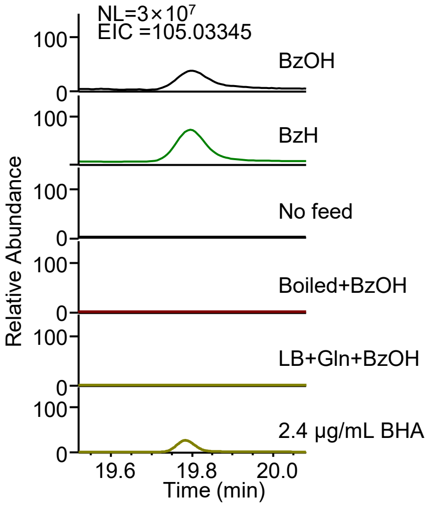

Figure S9. Detection of BHA in *E. coli* cultures heterologously expressing SaAmOx-AS, SaAmOx, and SaHAT after precursor feeding. Gln: Glutamine; BzH: Benzaldehyde; BzOH: Benzoic acid.

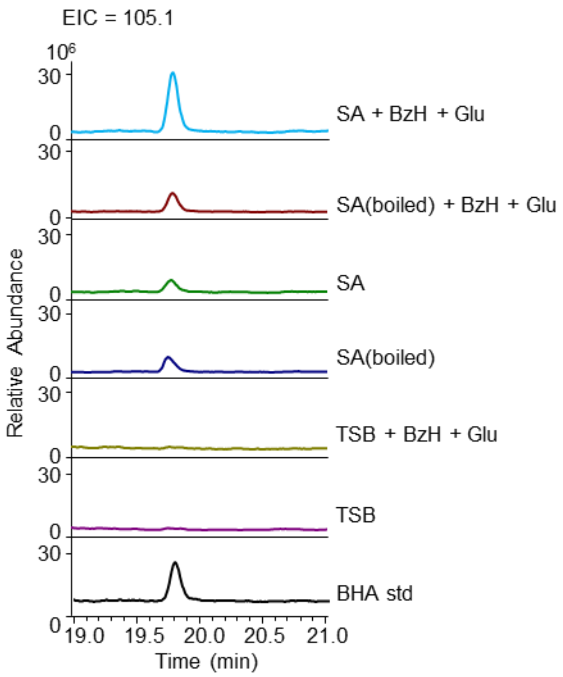

Figure S10. Representative GC-MS analysis of BHA production in *S. angustmyceticus*. The traces correspond to extracts from: (i) *S. angustmyceticus* cultures fed with benzaldehyde (BzH); (ii) heat-inactivated (boiled) *S. angustmyceticus* cultures fed with BzH; (iii) wild-type *S. angustmyceticus* (untreated); (iv) heat-inactivated wild-type *S. angustmyceticus*; (v) cell-free culture medium supplemented with BzH; and (vi) cell-free culture medium.

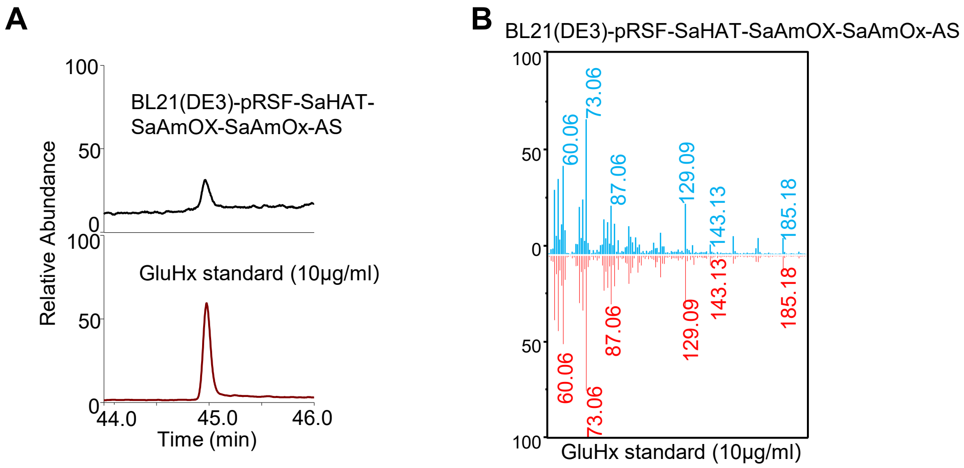

Figure S11. Gas chromatography-mass spectrometry detection results of GluHx.

A. Gas chromatographic comparison of GluHx of BL21(DE3)-pRSF-SaHAT-SaAmOx-SaAmOx-AS with standards. B. Comparison of the GluHx peak at 44.98 min of BL21(DE3)-pRSF-SaHAT-SaAmOx-SaAmOx-AS with the standard.
